# Fear of COVID-19 Among College Students: A Systematic Review and Meta-Analysis

**DOI:** 10.3389/fpubh.2022.846894

**Published:** 2022-03-01

**Authors:** Fang Wang, Le Zhang, Lu Ding, Lei Wang, Yang Deng

**Affiliations:** ^1^School of Public Health, Shandong First Medical University & Shandong Academy of Medical Sciences, Tai'an, China; ^2^Department of Public Health, Second Affiliated Hospital of Shandong First Medical University, Tai'an, China; ^3^School of Pharmaceutical Sciences, Shandong First Medical University & Shandong Academy of Medical Sciences, Tai'an, China

**Keywords:** fear, COVID-19, college students, meta-analysis, mental health

## Abstract

**Background:**

Mental health issue among college students is routinely a major public health concern, and coronavirus disease 2019 (COVID-19) pandemic may have exacerbated the students' mental health issues which include psychological distress, panic disorder, insomnia, and posttraumatic stress symptoms. However, few studies reached a consensus on the impact of COVID-19 fear on mental health among college students. Therefore, we aimed to conduct a systematic review and meta-analysis that quantitatively synthesized the fear among college students during the COVID-19 pandemic.

**Methods:**

PubMed, EMBASE, CINAHL, and PsycINFO electronic databases were systematically searched to identify cross-sectional study reporting the state of COVID-19 fear examined by the Fear of COVID-19 Scale (FCV-19S) published up until November 20, 2021. Methodological quality was complied with the evaluation criteria of the Agency for Healthcare Research and Quality. The random effects model was employed to estimate the pooled mean of FCV-19S score. Subgroup analysis and meta-regression analysis were also conducted. Publication bias was assessed by Begg's test and funnel plot.

**Results:**

A total of 16 studies with a sample size of 11,872 were included. A pooled mean of FCV-19S score was 17.60 [95% confidence interval (CI): 16.41–18.78]. The mean of COVID-19 fear in women (17.11, 95% CI: 16.59–17.64) was higher than that in men (15.21, 95% CI: 14.33–16.08). The highest and lowest pooled means of FCV-19S score were observed in the studies conducted in multiple countries that include Israel, Russian, and Belarus (21.55, 95% CI: 20.77–22.33) and in Europe (16.52, 95% CI: 15.26–17.77), respectively. No significant publication bias was detected by Begg's test.

**Conclusions:**

College students experienced a moderate level of fear caused by COVID-19 pandemic. It is necessary to design and implement prevention programs that target the mental health of college students.

**Systematic Review Registration:**

https://www.crd.york.ac.uk/prospero/display_record.php?ID=CRD42021287017, identifier: CRD42021287017.

## Introduction

Globally, the coronavirus disease 2019 (COVID-19) pandemic has led to over 249 million confirmed cases and more than 5 million deaths as of November 20, 2021 (1). In the early stages of COVID-19 pandemic, myths and misinformation driven by erroneous reports and misunderstanding of COVID-19 dramatically concerned the public (2, 3). Due to the high risk of infection and high fatality rate, the COVID-19 pandemic has caused public panic and predisposed individuals to deterioration in mental health (4). To prevent the spread of this health catastrophe, many countries and regions have implemented strict prevention and control measures, such as home quarantine, social distancing, compulsory face mask usage, and community-wide containment (5). However, these measures have forced a large number of people to keep away from the normal life. Actually, previous studies reported that COVID-19 pandemic and dramatic changes in people's daily lives posed substantial threats to the public's physical and mental health such as feeling fears and anxiety (6, 7). Persistent fear can trigger a series of physical functional disorders, for instance, chest pain, chest tightness, shortness of breath, palpitation, fatigue, and insomnia, and further progression may cause the occurrence of depression, anxiety, stress disorders, and endocrine disorders (6–8).

Fear is defined as an unpleasant emotional state that is elicited by the perception of danger and insecurity feelings (9). Fear, as one of the most factors that generate emotional issues such as anxiety and stress disorders, is responsible for the impairment of mental health (10). Previous studies revealed that pandemics that include severe acute respiratory syndrome, Ebola, and Middle East respiratory syndrome led to an increased feeling of fear among the population (11, 12). Essentially, the impacts of pandemics on fear are mainly reflected in two aspects. First, fear is directly associated with high transmission rate and rapid and invisible medium of infectious diseases caused by the virus. Second, fear is an indirect consequence of quarantine and other control measures (13). Recent studies declared that COVID-19 was a determinant of fear, and fear caused healthcare seeking delays or even increasing suicide rate during the pandemic (14, 15). It is essential to determine the important role of fear caused by COVID-19 in understanding the impact of pandemic on mental health and implementing appropriate interventions. Based on this, Ahorsu et al. developed the Fear of COVID-19 Scale (FCV-19S) which was a brief and valid instrument to detect an individual's fear of COVID-19 (16). The FCV-19S has been validated in several countries and translated into multiple languages that include English, Italian, Bangla, Turkish, Vietnamese, Japanese, Spanish, Russian, and Chinese (13, 16–26). The FCV-19S is a questionnaire consisted of seven items. These seven items were designed according to an extensive review of all existing scales on fears such as the Hospital Anxiety and Depression Scale and Perceived Vulnerability to Disease Scale, and then, they were evaluated by expert panels and interviewed by participants (16). Each item is evaluated on a 5-piont Likert scale, from 1 (strongly disagree) to 5 (strongly agree). The score of each item ranges from 1 to 5, and the minimum and maximum scores for the overall items are 7 and 35, respectively. A higher score indicates a greater fear of COVID-19. Due to no official severity for FCV-19S being provided, we used a severity scale using percentiles of FCV-19S score as follows: mild (<17), moderate (18 to 23), and severe (more than 24) (17). The original version of FCV-19S showed strong psychometric qualities that include satisfactory internal consistency (Cronbach's α = 0.82) and test–retest reliability (intraclass correlation coefficient = 0.72) (16).

Compared to the general population, college students are reported to be more vulnerable to sudden changes in COVID-19 pandemic (27). Students are required to be confined in the family home due to shutdowns of many universities, and social distancing and self-isolation restricts their interaction with teachers and colleagues. Face-to-face teaching has shifted to online courses to avoid a physical meeting of teachers with students, whereas difficulty in adapting to online courses makes the students feel more fear and anxiety (28). Furthermore, Ahorsu et al. reported that COVID-19-related variables (e.g., fear) were associated with suicidal ideation and anxiety among college students (29). Similar findings have been reported by Pramukti et al. (30). Additionally, Sharma et al. have reported the high levels of psychological distress among college students during COVID-19 pandemic (31). In this study, we aimed to assess the fear of COVID-19 among college students, which may help implement psycho-educational interventions at the university level to alleviate the impacts of the pandemic on mental health.

## Materials and Methods

This systematic review and meta-analysis were conducted in accordance with the Preferred Reporting Items for Systematic Reviews and Meta-Analyses (PRISMA) guidelines (32) (Supplementary Table 1), and the protocol was registered in the International Prospective Register of Systematic Reviews (PROSPERO) with the registration number CRD 42021287017 (available from https://www.crd.york.ac.uk/prospero/display_record.php?ID=CRD42021287017). In this systematic review and meta-analysis, we aimed to estimate the pooled mean of fear of COVID-19 among college students.

### Search Strategy

We systematically searched PubMed (2019–2021), EMBASE (2019–2021), CINAHL (2019–2021), and PsycINFO (2019–2021) electronic databases to identify such literatures published up until November 20, 2021. The search terms were “2019 novel coronavirus-infected pneumonia” or “2019 novel coronavirus” or “2019 novel coronavirus pneumonia” or “COVID-19 pneumonia” or “COVID-19” or “2019-nCOV,” “undergraduate” or “university student” or “college student” or “higher education student,” and “anxiety” or “worry” or “fear” or “concern.” The detailed search strategy is shown in Supplementary Table 2. Two investigators (FW and YD) independently performed the literature searching and screened the retrieved studies. Moreover, the reference lists of included studies were manually screened to identify additional studies.

### Selection Criteria

The inclusion criteria were as follows: (1) cross-sectional study; (2) the state of fear of COVID-19 was examined by the FCV-19S; (3) the mean and standard deviation of FCV-19S score were reported; (4) the study was conducted among college students; and (5) publications published in English. Studies were excluded based on the following criteria: (1) studies without sufficient data; (2) publication of review, conference summary, and other non-original research; (3) multiple participants that did not present results separately for college students; and (4) methodological quality score (<4).

### Data Collection and Quality Assessment

Two independent investigators (FW and LD) screened the articles and selected those having the search terms in their titles or abstracts. Full-text articles which initially met the selection criteria were included. The kappa coefficient was calculated for measuring the agreement between two investigators making inclusion or exclusion decision. Disagreements between two investigators were resolved through negotiation with a third researcher (LZ). Then, they extracted the following data using a predesigned electronic form. The following data included the name of first author, publication year, age, gender, sample size, country of participants, and mean and standard deviation of FCV-19S score. According to the evaluation criteria for an observational study of the Agency for Healthcare Research and Quality (AHRQ), the quality assessment of the included studies was performed by two independent investigators (FW and LD) and assigned the methodological quality score. AHRQ contains 11 items, and each item is answered as yes, no, or not reported. The score of the answer “yes” is 1, whereas “no” or “not reported” scores 0. In terms of methodological quality score, studies were defined as low (0 to 3), moderate (4 to 7), and high qualities (8 to 11) (33). Thus, we incorporated the studies which were defined as moderate and high quality into the meta-analysis.

### Statistical Analysis

Meta-analysis was employed to estimate the pooled raw mean of FCV-19S score and the corresponding 95% confidence interval (CI). We used Cochran's *Q* test and *I*^2^ statistics to detect the heterogeneity among eligible studies. A fixed effects model was applied to generate the pooled raw mean when few evidence of heterogeneity was found among these studies (*I*^2^ ≤ 50% and *p* for heterogeneity ≥ 0.10); otherwise, the random effects model was used. The forest plot was adopted to visually depict the heterogeneity among the studies and show the pooled raw mean of FCV-19S score of selected studies. Subgroup analyses by continent (Asia/Europe/America/Multiple), publication year, and gender were conducted to examine the source of heterogeneity. Meta-regression method was employed to evaluate the association of raw mean of FCV-19S score with mean age and sample size of participants in included studies. Leave-one-out sensitivity analysis by dropping out one study at a time to estimate the pooled raw mean of remaining studies was conducted to determine whether a single study was a significant source of heterogeneity. Publication bias was assessed by Begg's test and funnel plot. The statistical analyses were performed using R software version 4.1.1 with meta and metaphor packages, and all statistical tests were two-tailed. A *p*-value < 0.05 was considered statistically significant except for the examination of heterogeneity.

## Results

### Literature Search and Study Selection

**Figure 1:**
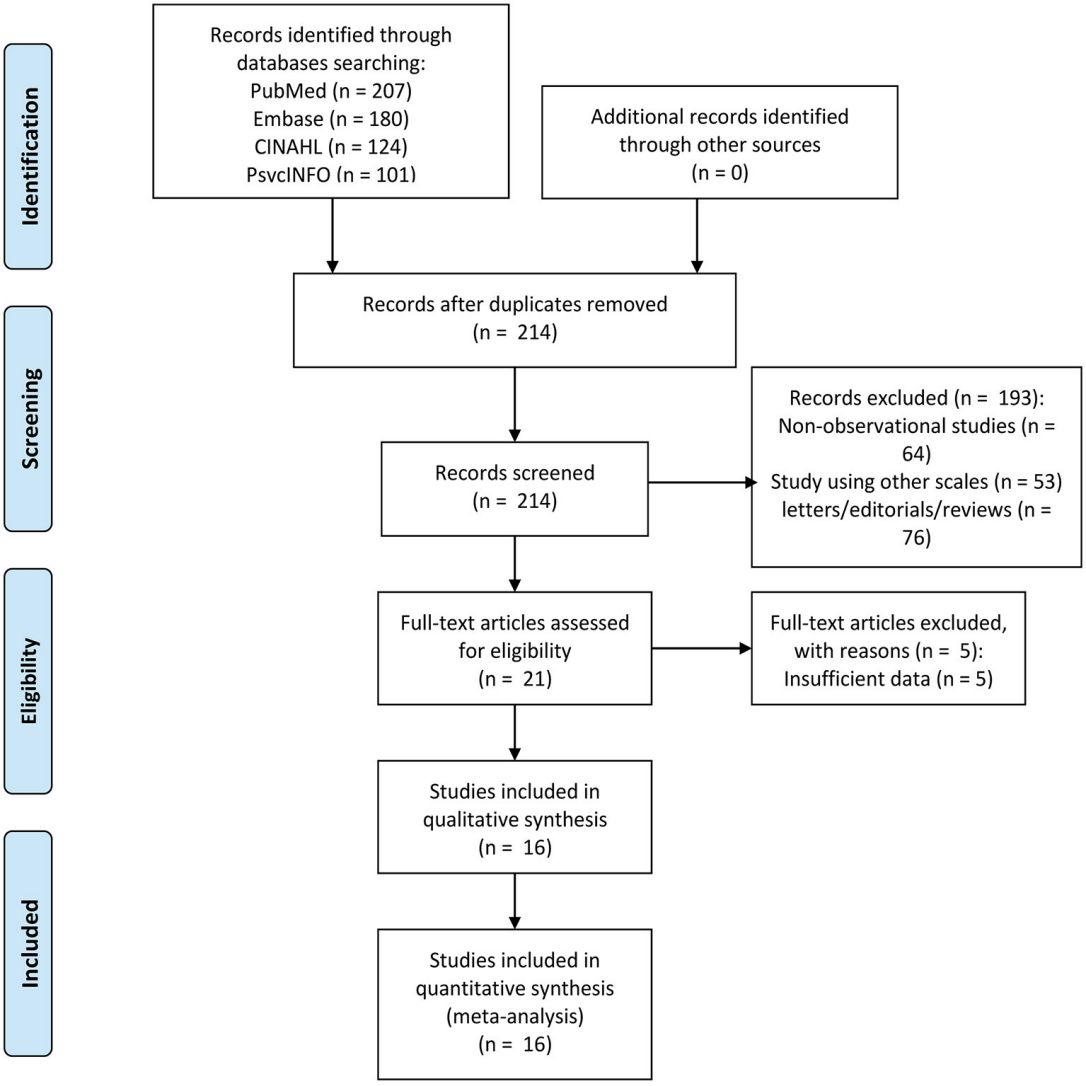
Flowchart of selecting and screening articles.

The flowchart of literature search and study selection based on the PRISMA guidelines is shown in Figure 1. A total of 612 articles were initially retrieved from PubMed, EMBASE, CINAHL, and PsycINFO electronic databases, of with 398 duplicate articles were removed. The titles and abstracts of remaining 214 articles were screened, and 193 articles were removed for the following reasons: non-observational studies (*n* = 64), study using other scales (*n* = 53), and letters–editorials–reviews (*n* = 76). In addition, 21 articles were eligible for the full-text screening, and 5 articles were excluded for insufficient data. Agreement between two independent investigators regarding the study selection was considered perfect (kappa coefficient = 0.79). Finally, 16 articles were included in the meta-analysis.

### Characteristics of Included Studies

As shown in Table 1, we included 16 studies with a sample size of 11,872 (4,733 men, 6,911 women, and 228 genders not mentioned). Among the included studies, 6 studies were from Asia (13, 21, 34–37), 6 from Europe (22, 23, 38–41), 2 from America (42, 43), and 2 from multiple continents (44, 45). All studies reported the total score of FCV-19S, 4 studies reported the corresponding score by gender (21, 37, 40, 41), and 5 studies reported the mean scores by item of FCV-19S (13, 36–38, 42). Moreover, the mean scores by item were reported in Fawzy El-Bardan's study (46), but the total score was not shown. The quality assessment of included studies revealed that eight studies had moderate quality (13, 21, 22, 34, 36, 37, 39, 40, 42–45), and other studies had high quality (23, 35, 38, 41).

**Table 1:** Characteristics of studies included in the meta-analysis.

**References**	**Sample size**			**Age (years)**	**Place**	**FCV-19S mean score**		
	**Total**	**Male**	**Female**			**Total**	**Male**	**Female**
Saravanan et al. (34)	433	278	155	21.00 ± 2.90	United Arab Emirates	16.60 ± 6.30	–	–
Rodríguez-Hidalgo et al. (23)	640	179	461	21.69 ± 4.09	Spain	14.37 ± 5.38	–	–
Nguyen et al. (21)	5,423	2,602	2,821	22.00 ± 2.00	Vietnam	16.70 ± 5.30	16.20 ± 5.60	17.00 ± 4.80
Martínez-Lorca et al. (38)	606	109	497	21.59 ± 3.04	Spain	16.79 ± 6.04	–	–
Konstantinov et al. (35)	466	154	312	19.00 ± 2.70	Kazakhstan	22.10 ± 5.80	–	–
Reznik et al. (22)	228	–	–	–	Russia and Belarus	18.00 ± 4.50	–	–
Zolotov et al. (36)	366	77	289	25.20 ± 3.10	Israel	14.95 ± 4.80	–	–
Perz et al. (42)	237	64	173	30.30 ± 10.20	USA	18.10 ± 7.10	–	–
Yehudai et al. (44)	288	46	242	24.40 ± 5.50	Israel and Russia	22.00 ± 6.30	–	–
Masuyama et al. (13)	629	320	309	–	Japan	18.71 ± 5.65	–	–
Isralowitz et al. (45)	593	173	420	–	Israel, Russia, and Belarus	21.20 ± 6.10	–	–
Hasratian et al. (43)	175	58	117	19.83 ± 1.74	USA	15.33 ± 5.75	–	–
De Pasquale et al. (39)	469	221	248	22.47 ± 2.70	Italy	15.90 ± 6.06	15.02 ± 6.02	16.68 ± 6.00
De Pasquale et al. (40)	194	86	108	21.74 ± 2.39	Italy	15.53 ± 6.01	14.01 ± 5.80	16.75 ± 6.24
Muyor-Rodríguez et al. (41)	517	108	409	21.03 ± 4.32	Spain	18.5 ± 5.88	–	–
Green et al. (37)	608	258	350	24.76 ± 3.52	Pakistan	16.74 ± 6.68	15.10 ± 5.88	17.94 ± 6.98

### Meta-Analysis of FCV-19S Score

**Figure 2:**
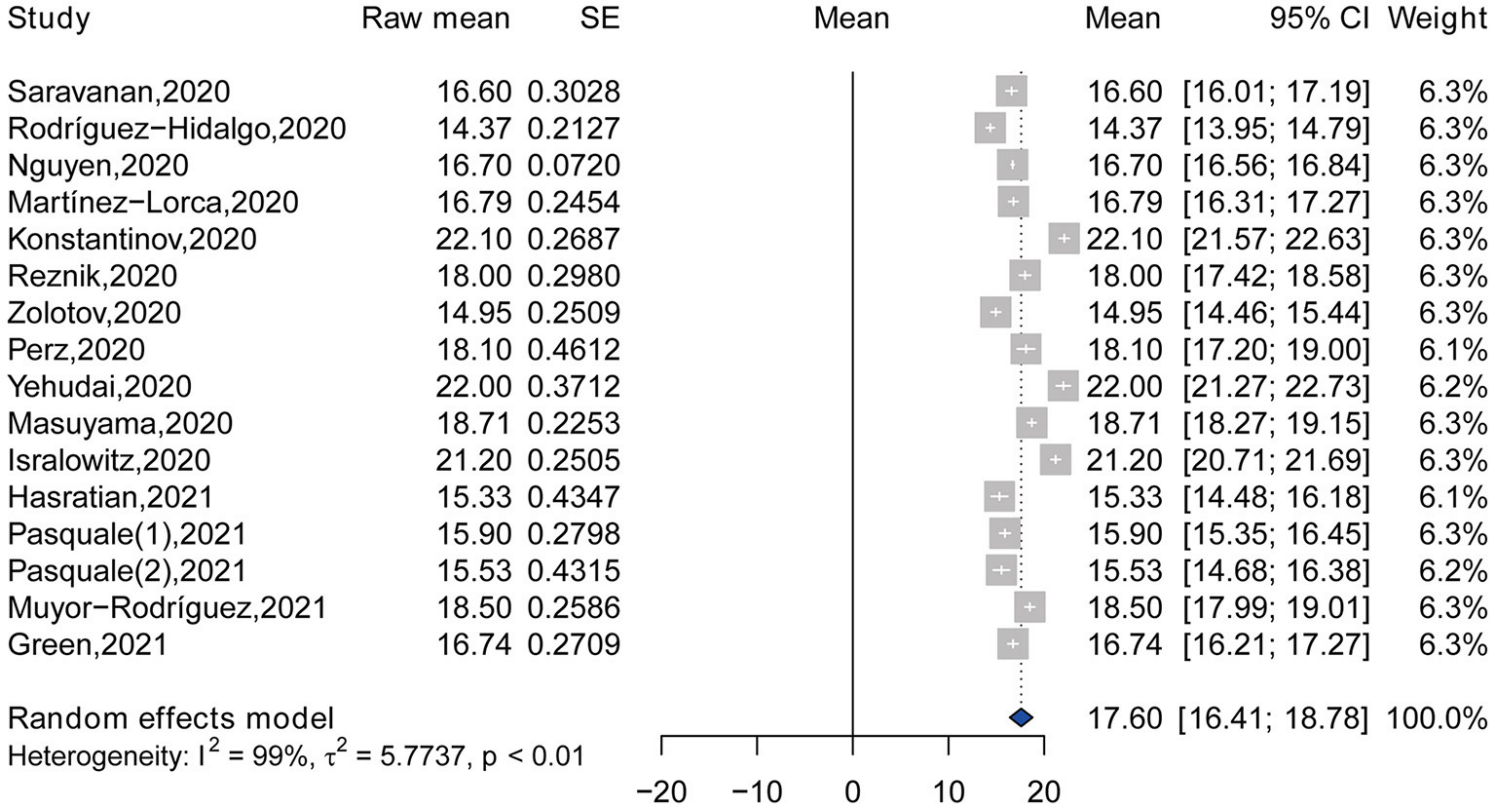
Forest plot showing the pooled mean of fear of COVID-19 (*n* = 16).

According to the level of heterogeneity among included studies, random effects model was used to estimate the pooled raw mean of FCV-19S score (*Q* = 1,205.27, df = 15, *p* < 0.01, τ^2^ = 5.7737, *I*^2^ = 98.8%). The pooled raw mean of FCV-19S score was 17.60 (95% CI: 16.41–18.78) (Figure 2), which suggests that a moderate level of fear was observed among college students during COVID-19 pandemic. Among the 6 studies reported the mean of FCV-19S score by item, the highest and lowest pooled raw means of FCV-19S score were observed in the item 1 (I am most afraid of coronavirus 19) (3.56, 95% CI: 2.82–4.30) and item 3 (my hands become clammy when I think about coronavirus 19) (1.78, 95% CI: 1.51–2.05), respectively (Table 2; Supplementary Figures 1–7).

**Table 2:** Pooled mean of fear of COVID-19 scale by item.

**Item**	**Mean**	**95% CI**
1. I am most afraid of coronavirus 19.	3.56	2.82–4.30
2. It makes me uncomfortable to think about coronavirus 19.	3.30	2.74–4.03
3. My hands become clammy when I think about coronavirus 19.	1.78	1.51–2.05
4. I am afraid of losing my life because of coronavirus 19.	2.90	2.08–3.73
5. When watching news and stories about coronavirus 19 on social media, I become nervous or anxious.	3.29	2.67–3.91
6. I cannot sleep because I am worrying about getting coronavirus 19.	1.79	1.48–2.10
7. My heart races or palpitates when I think about getting coronavirus 19.	2.19	1.52–2.87

### Subgroup Analysis

**Figure 3:**
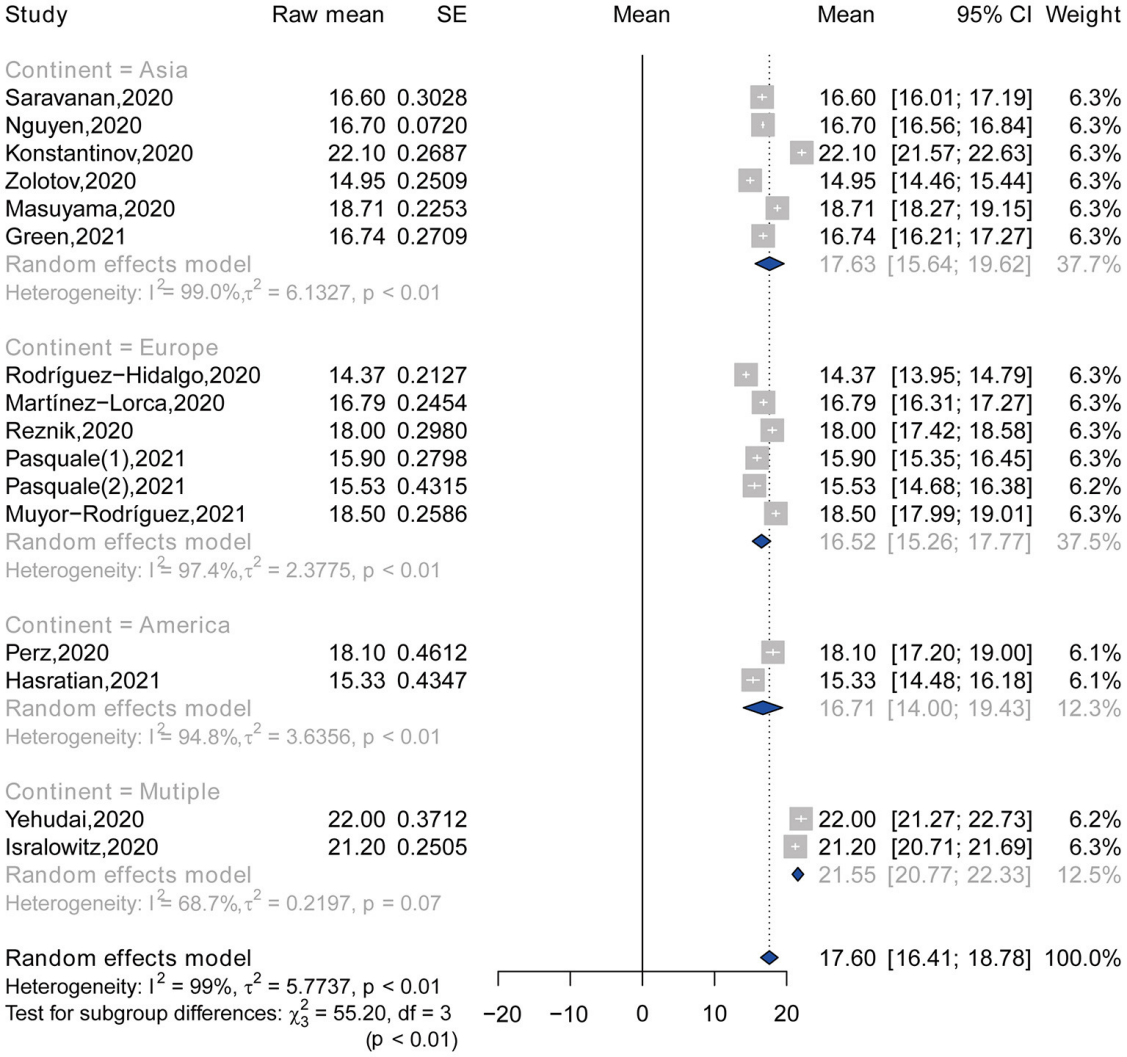
Forest plot of the total mean score of fear, according to continent.

**Figure 4:**
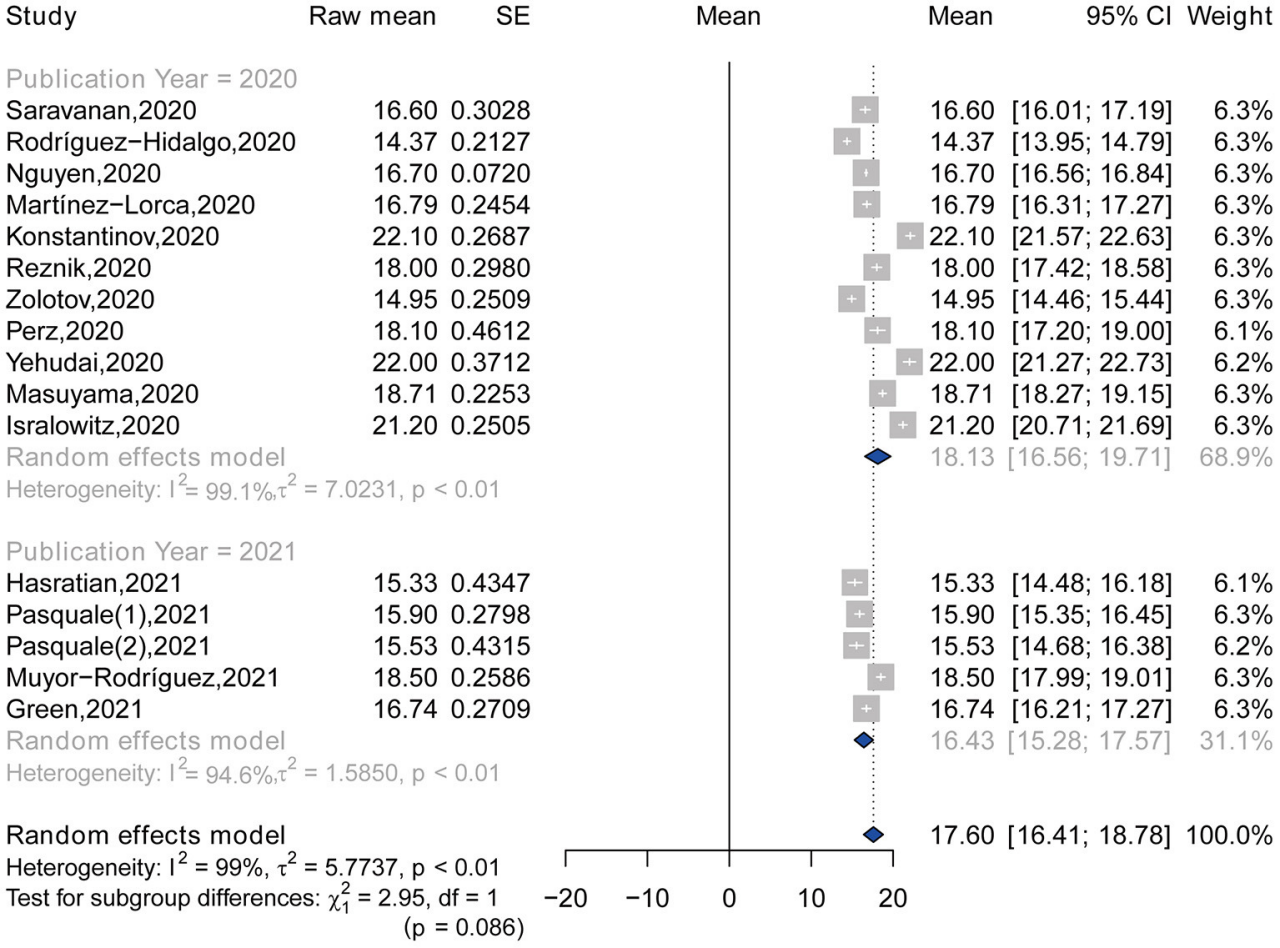
Forest plot of the total mean score of fear, according to publication year.

The highest and lowest pooled means of FCV-19S score were found in the studies conducted in multiple countries that include Israel, Russian, and Belarus (21.55, 95% CI: 20.77–22.33) and in Europe (16.52, 95% CI: 15.26–17.77), respectively (Table 3; Figure 3). No significant difference was observed in the pooled means of FCV-19S score between studies published in 2020 (18.13, 95% CI: 16.56–19.71) and in 2021 (16.43, 95% CI: 15.28–17.57), with a *p*-value of 0.086 (Table 3; Figure 4). Moreover, the pooled mean of FCV-19S score in men (15.21, 95% CI: 14.33–16.08) was lower than that in women (17.11, 95% CI: 16.59–17.64) (Supplementary Figure 8).

**Table 3:** Subgroup analysis of the pooled mean of fear of COVID-19 by continent and publication year.

**Characteristic**	**Group**	**Number**	**Pooled mean**	**95% CI**	**Heterogeneity**	**Subgroup differences test**		
					***I^**2**^* (%)**	***Q***	**df**	***p***
Continent	Asia	6	17.63	15.64–19.62	99.0	55.20	3	<0.001
	Europe	6	16.52	15.26–17.77	97.4			
	America	2	16.71	14.00–19.43	94.8			
	Multiple	2	21.55	20.77–22.33	68.7			
Publication year	2020	11	18.13	16.56–19.71	99.1	2.95	1	0.086
	2021	5	16.43	15.28–17.57	94.6			

The univariate meta-regression analysis revealed that the associations of raw means of FCV-19S score with mean age and sample size of participants were not statistically significant (*p* = 0.992; *p* = 0.738) (Supplementary Figure 9; Supplementary Table 3).

### Sensitivity Analysis and Publication Bias

**Figure 5:**
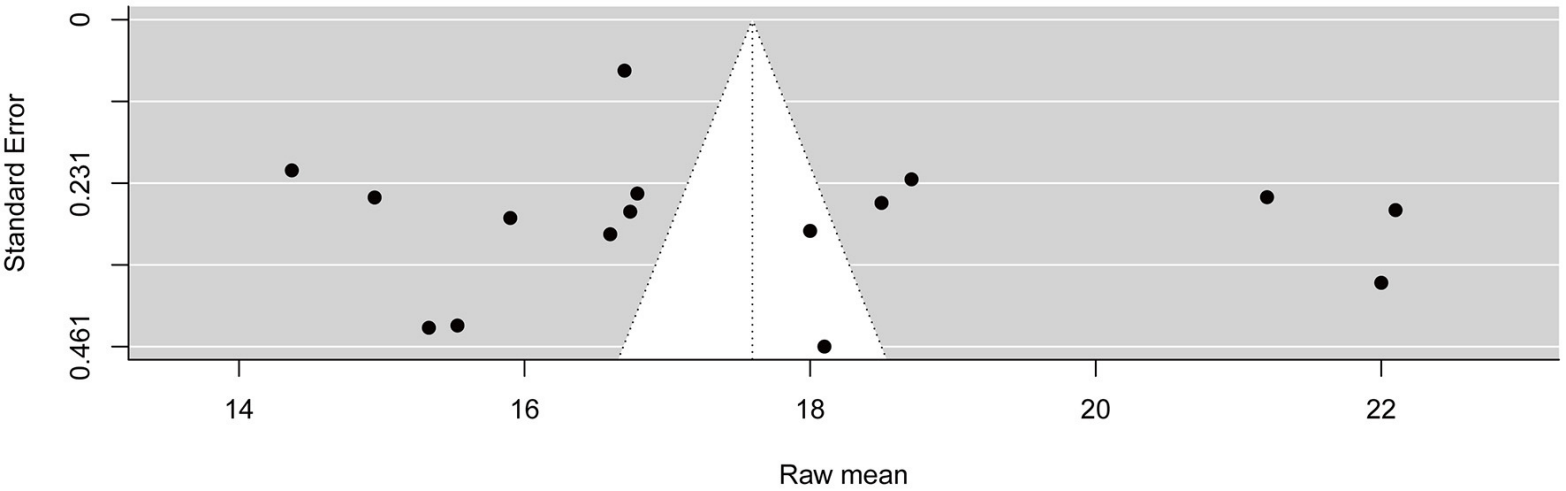
Funnel plot of publication bias based on Egger's regression test.

As shown in Supplementary Figure 10, the results of sensitivity analysis based on random effects model suggested that no individual study had a substantial influence on the pooled raw mean of FCV-19S score. In addition, Begg's test indicated that no publication bias was detected in this meta-analysis (*p* = 0.910) (Figure 5).

## Discussion

The present systematic review and meta-analysis included 16 studies with 11,872 participants. Our results showed that the pooled mean of FCV-19S score was 17.60 (95% CI: 16.41–18.78), which was lower than those in the general population and medical staff reported in a study by Luo et al. (47). Fear is regarded as one of the main contributors to anxiety and depression in the pandemic, whereas knowledge, attitudes, and practice of COVID-19 are the influencing factors of anxiety and depression. Zhong et al. also showed that people with better knowledge about COVID-19 are more optimistic about the controllability of the pandemic and therefore are more prepared to follow instructions for the prevention of its spread (48). A study conducted among medical students in Serbia revealed that students with sufficient knowledge had two times higher likelihood to wear masks outside compared to those with insufficient knowledge. Furthermore, sufficient knowledge was negatively associated with the FCV-19S score (49). Traditional media (e.g., television, newspapers, and radio), Internet, and social media have been demonstrated to be effective tools for the dissemination and application of new information and knowledge about COVID-19 (50). Compared to the general population, college students who spend much time searching and obtaining information about COVID-19 on these media channels may acquire more knowledge about the conception and prevention of COVID-19 (51). Furthermore, students with backgrounds in medical and health science have no more knowledge and fear than those without medical and health science backgrounds (52). This may be due to the broad coverage of updated information on COVID-19 in different media sources and strict lockdown measures enforced by governments. Thus, it is necessary for the governments to come up with good solutions to deliver information and control measures of COVID-19.

The pooled mean of FCV-19S score was higher in women than in men, which indicates that women experienced more fear during COVID-19 pandemic than men. This finding is consistent with the studies performed among the general population in Cuba and Bangladesh (19, 53) and among college students in Vietnam and Italy (20, 32, 33). This is probably attributed to women who are more susceptible to health risks associated with COVID-19 than men (53). In addition, compared to men, women are more emotionally vulnerable to COVID-19 pandemic in the field of exhibiting significantly higher levels of stress, insomnia, anxiety, and depression (54, 55). Women have been reported to perceive more risk of COVID-19 and suffer from more severe posttraumatic stress symptoms than men during the pandemic, which is mainly because women are more active in the neural networks related to fear than men (30). Women without social and emotional support were more vulnerable to the negative effect on mental health outcomes than men without social intercourse, because women were more reliant on social and emotional support. Women cannot get social and emotional support as before due to the strict lockdown during COVID-19, thus they feel the unprecedented effects of loneliness and isolation (56). In respect of college students, female students suffer more from stress and show lower stress coping abilities than male students (57). Thus, gender differences of COVID-19 fear among college students should be recognized. Policymakers need to keep in mind that the pandemic has distinct effects on the mental health of male and female students and roll out more targeted measures to tackle this issue.

This study found that the highest and lowest pooled means were observed in the items 1 and 3 of FCV-19S, respectively. This is likely attributed to what the items evaluate. Item 1 (I am most afraid of coronavirus 19) mainly assesses the attitude toward COVID-19, whereas item 3 (my hands become clammy when I think about coronavirus 19) especially refers to the hand symptoms of fear related to COVID-19. Similarly, participants received a score at the low level in item 6 (I cannot sleep because I am worrying about getting coronavirus 19). Both items 3 and 6 ask about symptoms of fear caused by COVID-19. Facing the spread of the COVID-19, a large number of people feel fear and anxiety, but only a small number of severely anxious individuals will have obvious symptoms that include sleepless and muscular tensing (58).

Since the first case of COVID-19 was reported in Wuhan, China in late 2019, the pandemic has left the world lasting scars in both disastrous mortality and public panic. The results of this study indicated that there was no significant difference in the FCV-19S scores between studies published in 2020 and 2021. Furthermore, the emergence of SARS-CoV-2 variants has triggered new waves in many countries (59, 60). It can be assumed that the ongoing global pandemic is not effectively controlled worldwide, and the negative effect of COVID-19 on the mental health of general population might last long. In addition, our results showed that the highest FCV-19S scores were observed in the studies conducted in Israel, Russian, and Kazakhstan, whereas the lowest scores were found in those studies conducted in Italy. This result might be associated with the incidence and mortality from COVID-19 and vaccine doses administered in the general population. For example, Russian, the Eastern Europe's worst-hit country, has reported nearly 10 million confirmed cases of COVID-19 with a death toll of 287, 180 as of December 13, 2021 (61). However, the vaccination coverage of COVID-19 remains low in Russian, and the numbers of total vaccine doses administered and persons fully vaccinated are below the global average.

Moreover, different prevalence of fear of COVID-19 among different countries can be attributed to cultural contexts and other contextual factors and differences in access to medical care and mental healthcare (40). For instance, mask-wearing, an effective measure to fight airborne pathogens including SARS-CoV-2, is often cast as the trigger of cultural clash and even cultural violence. It is worth mentioning that Chinese actively react to the appeals for mask-wearing all along. China is regarded as one of the first countries to emphasize the benefit of mask-wearing among residents in the early stage of COVID-19 pandemic. Since the outbreak of COVID-19, some medical supplies were still in short supply, of which masks were the scarcest material in all frontline medical consumables, and China had the highest percentage of people wearing masks (62). It was estimated that more than 500 million masks were consumed per day when work resumed at half capacity. However, in the United States, governments in several states such as Wyoming, South Dakota, and Utah had no statewide mask mandates. The government of Sweden steadfastly resisted mask-wearing tactics, and hundreds of people gathered in Portugal's capital Lisbon to protest against the use of masks, appealing for the so-called freedom and truth (63).

Several limitations should be acknowledged in this study. First, studies included in the meta-analysis were cross-sectional studies, which reported the prevailing situation. Longitudinal studies are needed to examine the causal relationship between the mental health consequences and COVID-19 fear. Second, the FCV-19S score is not a clinically relevant value to be targeted by an intervention. However, the results of this study showed that college students experienced a moderate level of fear related to COVID-19. Third, only articles published in English were included in this meta-analysis, and qualified articles published in other languages were not included, which might affect the results.

In conclusion, the results of the present systematic review and meta-analysis showed that college students have experienced a moderate level of fear caused by COVID-19, with female students having higher level of fear than male ones. Disparity in the prevalence of fear of COVID-19 among countries might be attributed to cultural contexts and access to healthcare services. It is necessary for educational authorities and universities to develop appropriate mental health interventions for the fear of COVID-19 among college students.

## Data Availability Statement

The original contributions presented in the study are included in the article/Supplementary Material, further inquiries can be directed to the corresponding author/s.

## Author Contributions

YD and FW designed the study protocol. FW, LZ, and LD conducted the literature search. FW, LZ, and YD retrieved and selected the articles. FW, LZ, and LW conducted data extraction. FW and LZ performed the statistical analysis of the data. FW and YD wrote the manuscript draft. YD and LZ supervised the study. All authors contributed to the article and approved the submitted version.

## Funding

This work was supported by the National Natural Science Foundation of Shandong Province (Grant No. ZR2017PH012). The funder had no role in study design, data collection and analysis, decision to publish, or preparation of the manuscript.

## Conflict of Interest

The authors declare that the research was conducted in the absence of any commercial or financial relationships that could be construed as a potential conflict of interest.

## Publisher's Note

All claims expressed in this article are solely those of the authors and do not necessarily represent those of their affiliated organizations, or those of the publisher, the editors and the reviewers. Any product that may be evaluated in this article, or claim that may be made by its manufacturer, is not guaranteed or endorsed by the publisher.
