# Supplementary material for: Fear of COVID-19 Among College Students: A Systematic Review and Meta-Analysis
Source: Front Public Health. 2022 Mar 1;10:846894. doi: 10.3389/fpubh.2022.846894 (PMC8921101; doi:10.3389/fpubh.2022.846894)
Supplement: Supplementary file 1 [file Data_Sheet_1.DOCX]

***Supplementary Materials***

**Fear of COVID-19 among college students: A Systematic Review and Meta-Analysis**

Fang Wang, Le Zhang^*^, Lu Ding, Lei Wang, Yang Deng^*^

**^*^Correspondence:**

Yang Deng: dengyang3417@126.com.

Le Zhang: sdzhangle@163.com


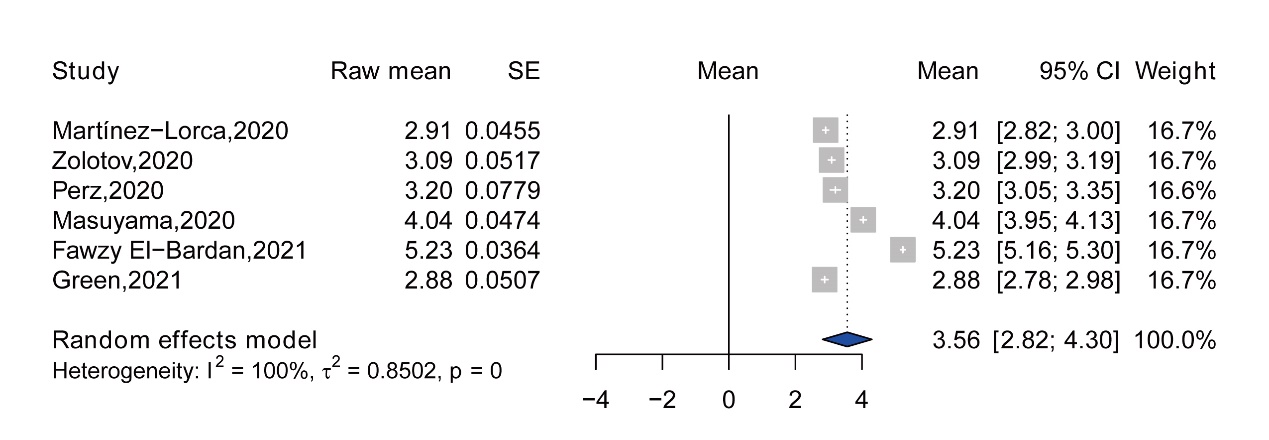


**Supplementary figure 1.** Forest plot of the fear Score mean of item 1


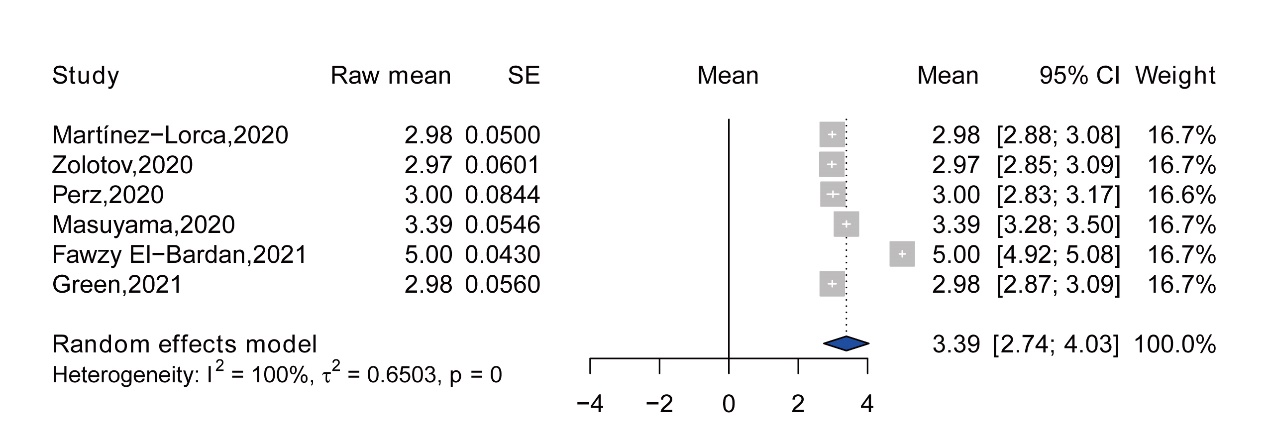


**Supplementary figure 2.** Forest plot of the fear Score mean of item 2


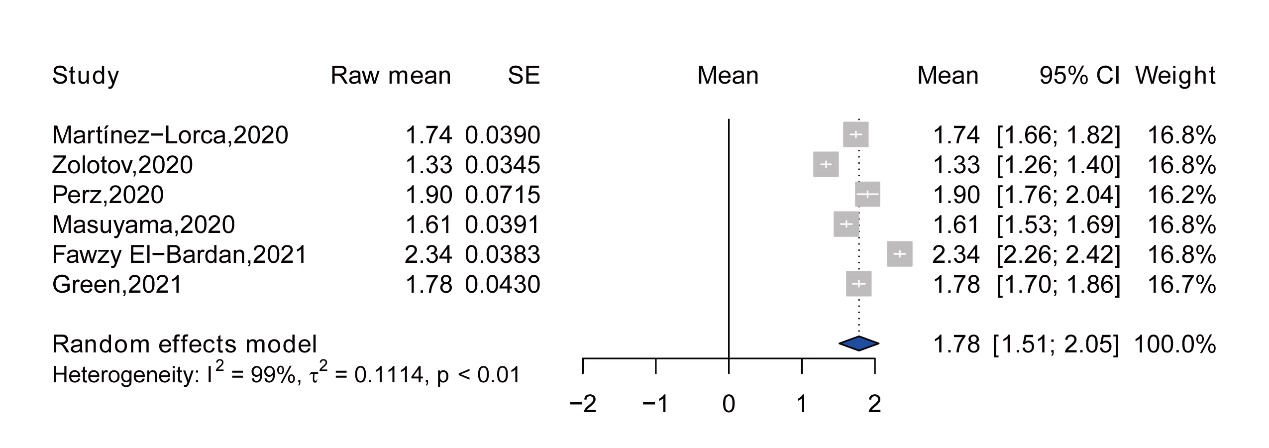


**Supplementary figure 3.** Forest plot of the fear Score mean of item 3


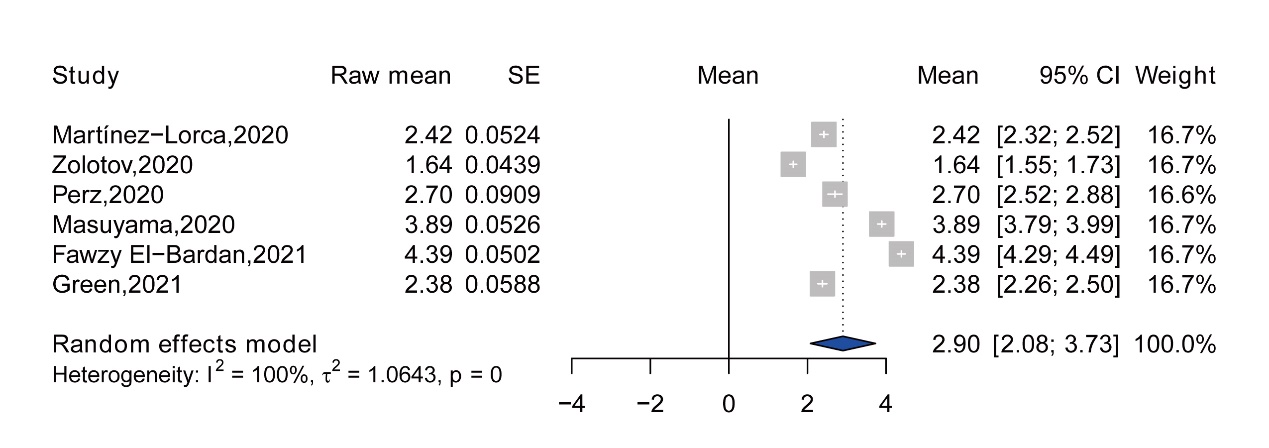


**Supplementary figure 4.** Forest plot of the fear Score mean of item 4


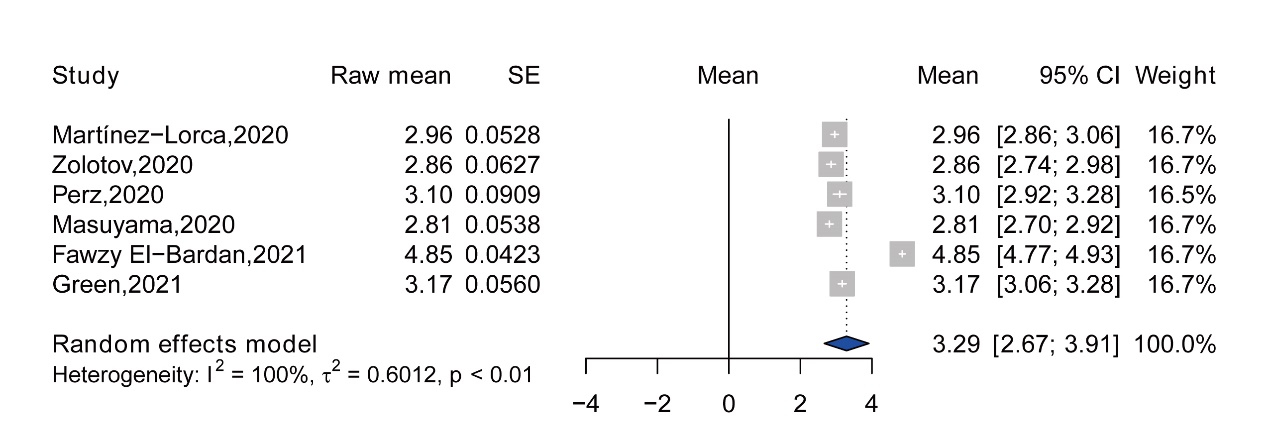


**Supplementary figure 5.** Forest plot of the fear Score mean of item 5


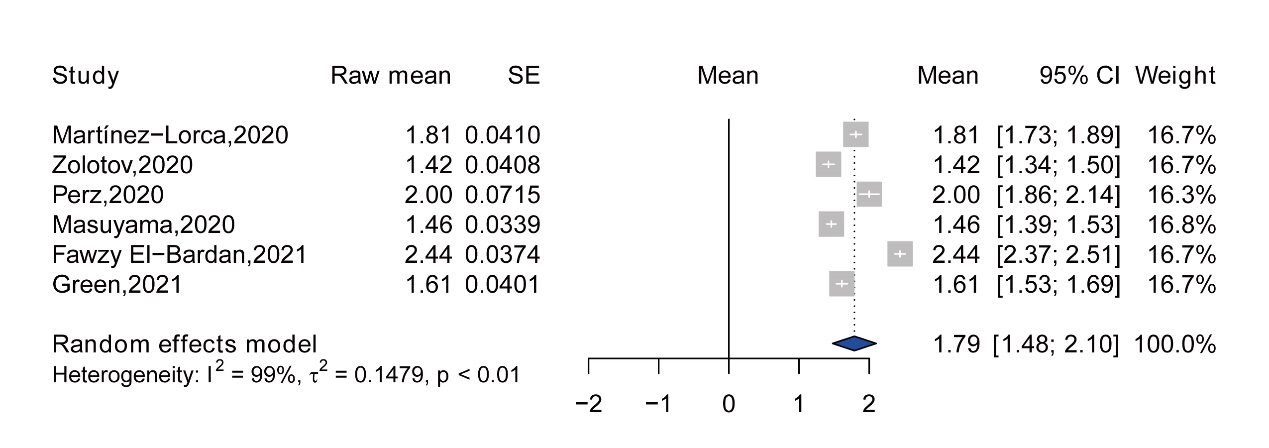


**Supplementary figure 6.** Forest plot of the fear Score mean of item 6


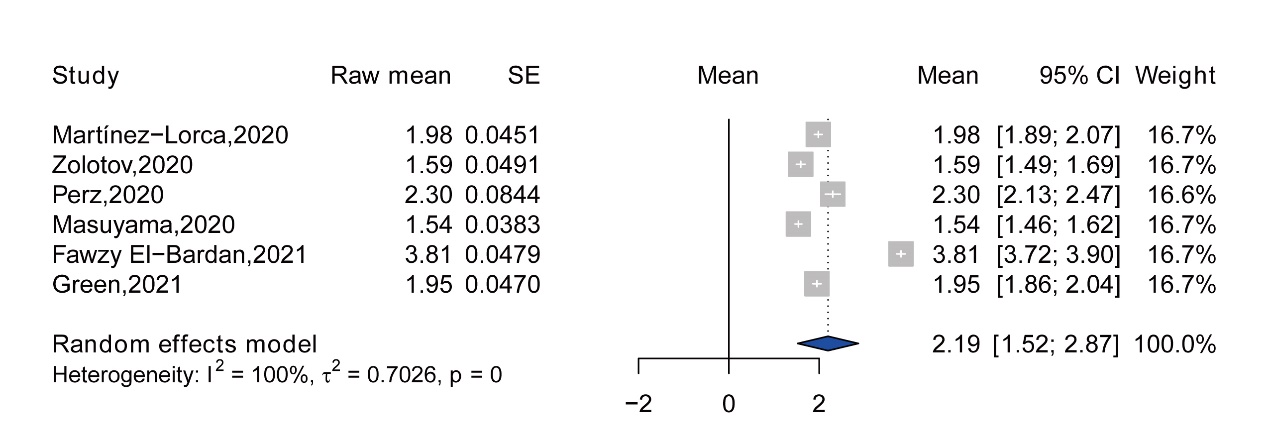


**Supplementary figure 7.** Forest plot of the fear Score mean of item 7

**A**
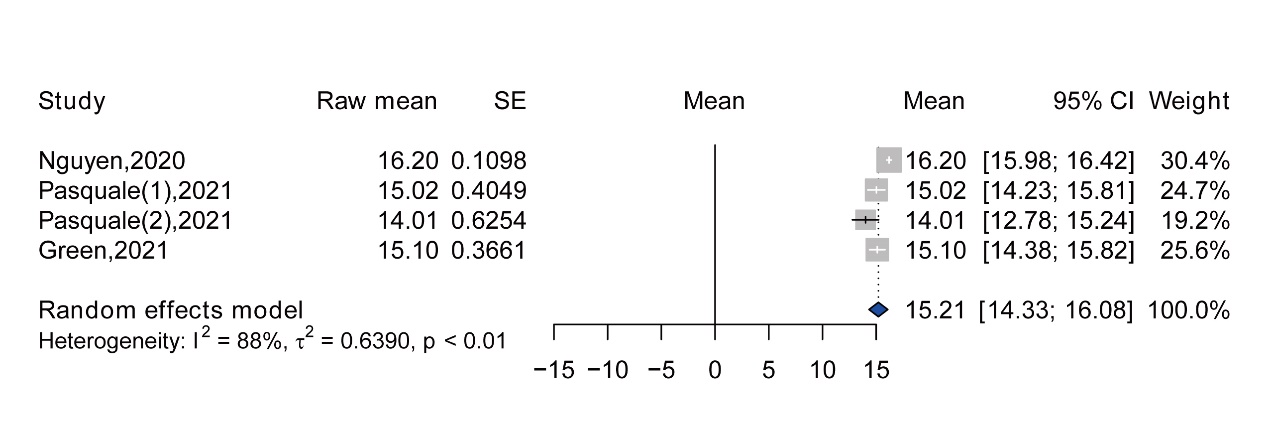


**B**

**
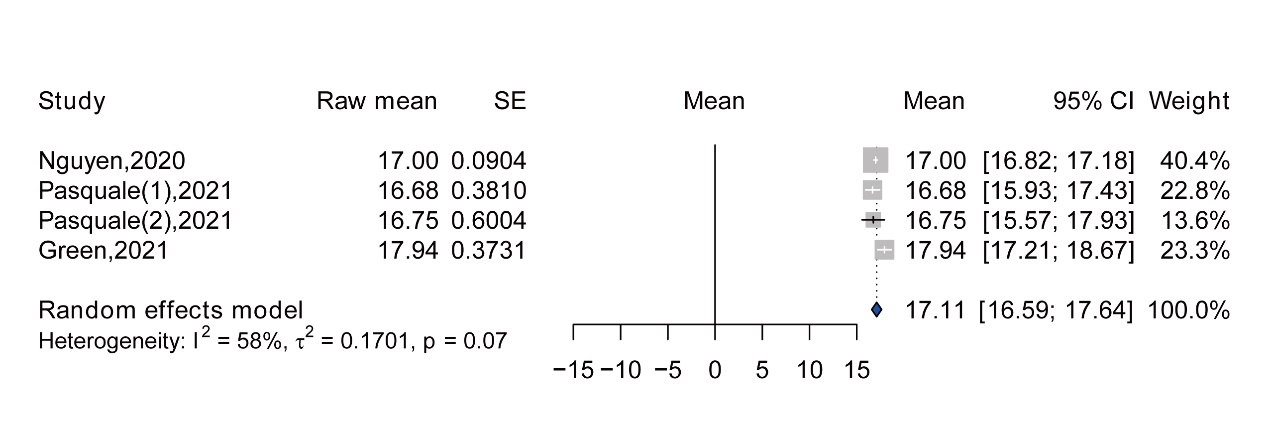
**

**Supplementary figure 8.** Forest plot of mean score of fear in men (A) and in women (B)

**A**

**
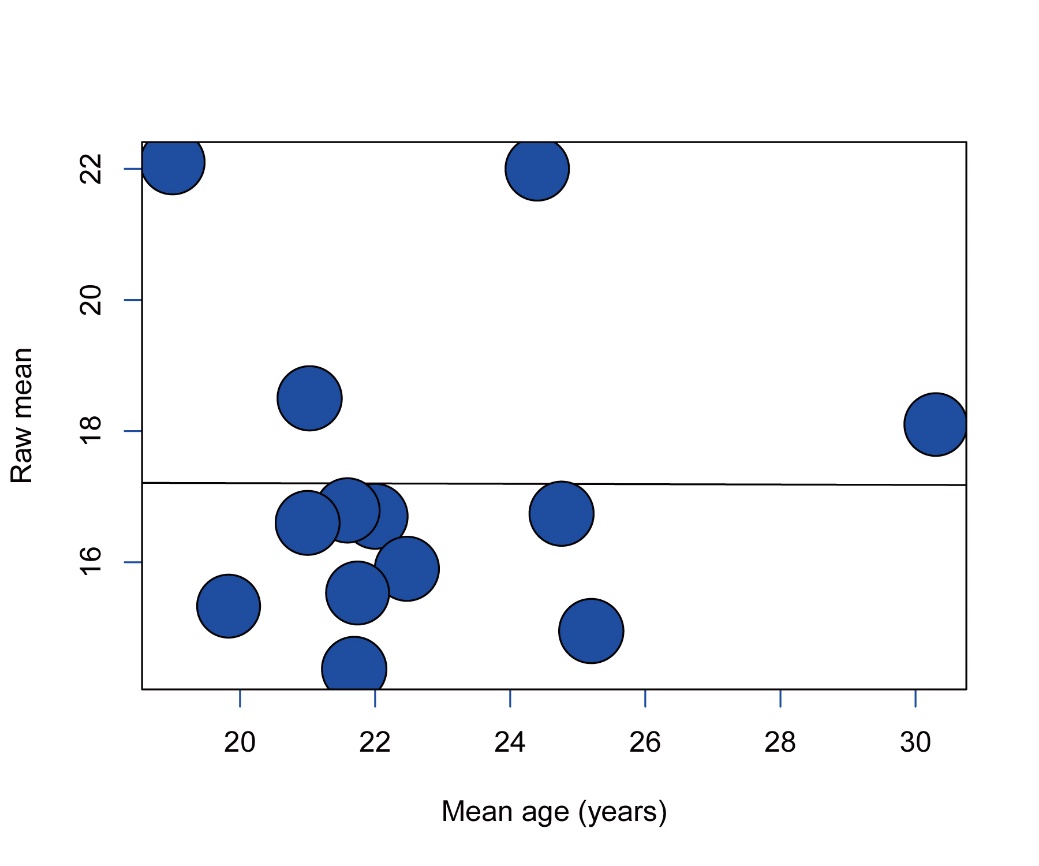
**

**B**

**
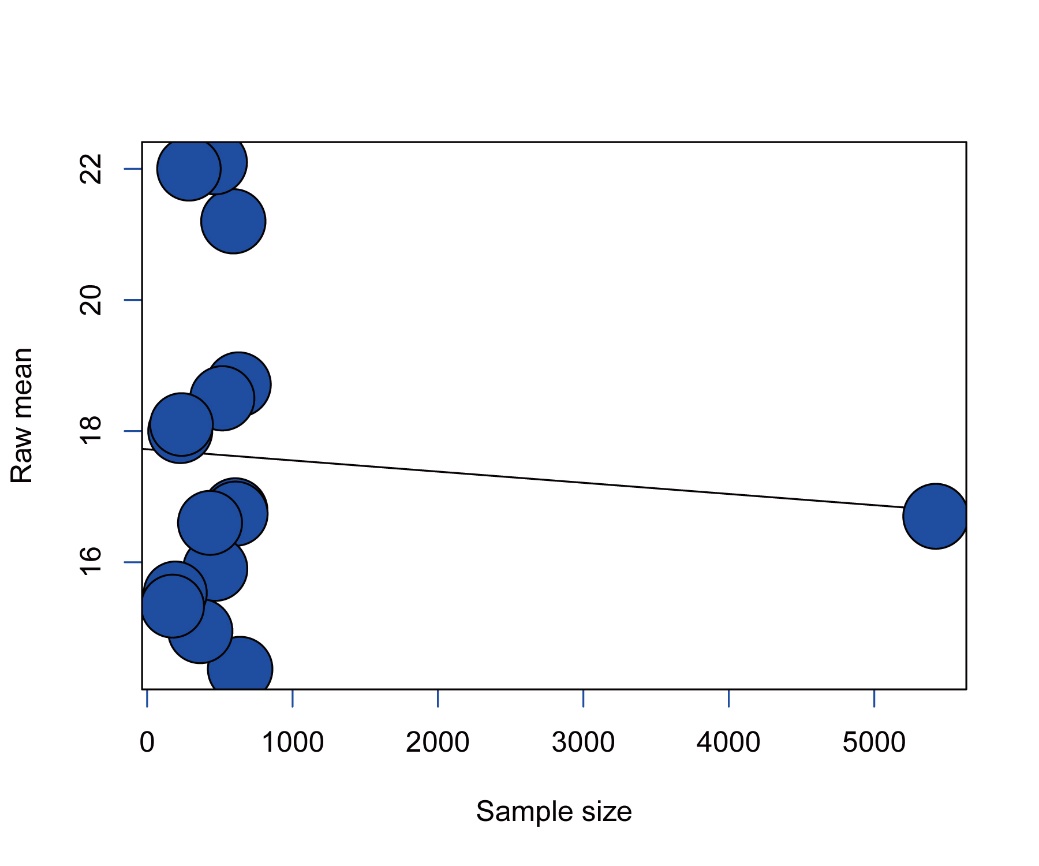
**

**Supplementary figure 9.** Meta-regression analysis of the relationship between mean of fear of COVID-19 and mean age of participants (A) and sample size (B)


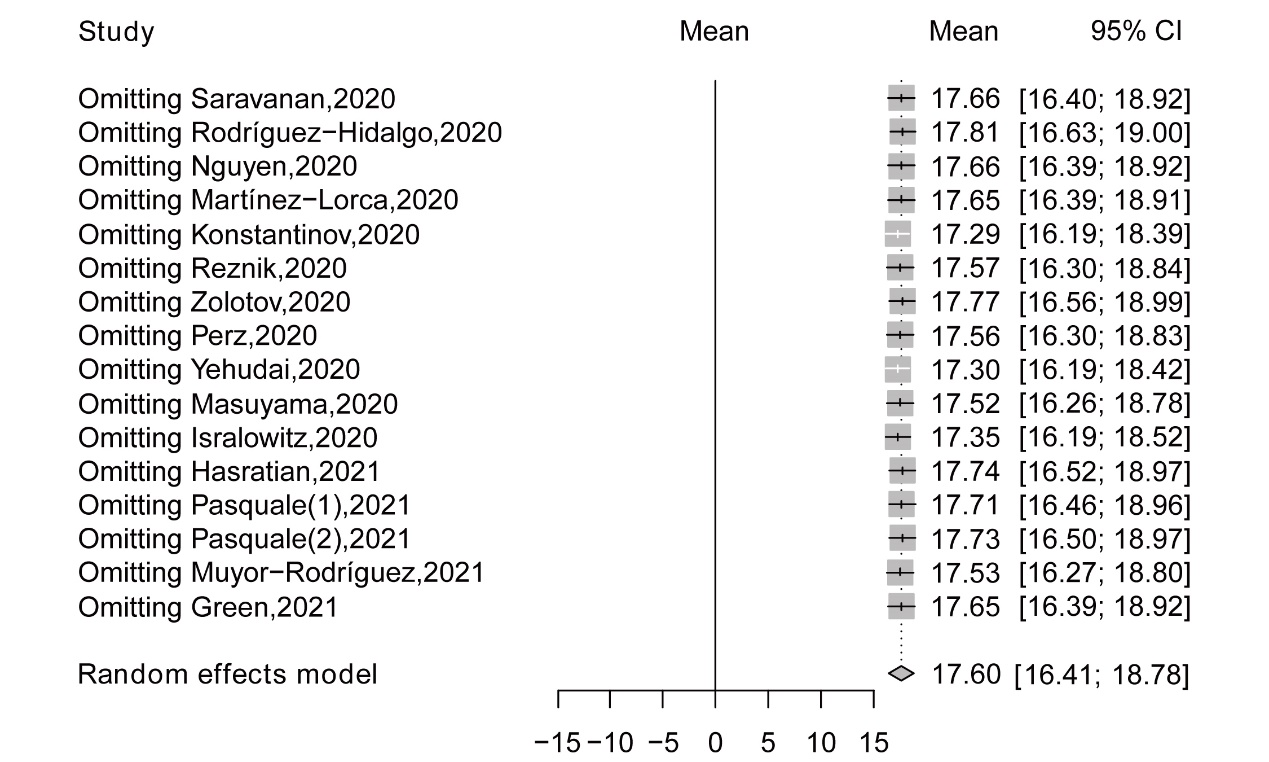


**Supplementary figure 10.** Plot of sensitivity analysis of total mean scores of fear based on leave-one-out method

**Supplementary Table 1.** PRISMA 2009 Checklist

| **Section/topic** | **#** | **Checklist item** | **Reported on page #** |
| --- | --- | --- | --- |
| **TITLE** | | |  |
| Title | 1 | Identify the report as a systematic review, meta-analysis, or both. | 1 |
| **ABSTRACT** | | |  |
| Structured summary | 2 | Provide a structured summary including, as applicable: background; objectives; data sources; study eligibility criteria, participants, and interventions; study appraisal and synthesis methods; results; limitations; conclusions and implications of key findings; systematic review registration number. | 2 |
| **INTRODUCTION** | | |  |
| Rationale | 3 | Describe the rationale for the review in the context of what is already known. | 4 |
| Objectives | 4 | Provide an explicit statement of questions being addressed with reference to participants, interventions, comparisons, outcomes, and study design (PICOS). | 4 |
| **METHODS** | | |  |
| Protocol and registration | 5 | Indicate if a review protocol exists, if and where it can be accessed (e.g., Web address), and, if available, provide registration information including registration number. | 6 |
| Eligibility criteria | 6 | Specify study characteristics (e.g., PICOS, length of follow-up) and report characteristics (e.g., years considered, language, publication status) used as criteria for eligibility, giving rationale. | 7 |
| Information sources | 7 | Describe all information sources (e.g., databases with dates of coverage, contact with study authors to identify additional studies) in the search and date last searched. | 7 |
| Search | 8 | Present full electronic search strategy for at least one database, including any limits used, such that it could be repeated. | 7 |
| Study selection | 9 | State the process for selecting studies (i.e., screening, eligibility, included in systematic review, and, if applicable, included in the meta-analysis). | 8 |
| Data collection process | 10 | Describe method of data extraction from reports (e.g., piloted forms, independently, in duplicate) and any processes for obtaining and confirming data from investigators. | 8 |
| Data items | 11 | List and define all variables for which data were sought (e.g., PICOS, funding sources) and any assumptions and simplifications made. | 8 |
| Risk of bias in individual studies | 12 | Describe methods used for assessing risk of bias of individual studies (including specification of whether this was done at the study or outcome level), and how this information is to be used in any data synthesis. | 9 |
| Summary measures | 13 | State the principal summary measures (e.g., risk ratio, difference in means). | 9 |
| Synthesis of results | 14 | Describe the methods of handling data and combining results of studies, if done, including measures of consistency (e.g., I^2^) for each meta-analysis. | 9 |

| **Section/topic** | **#** | **Checklist item** | **Reported on page #** |
| --- | --- | --- | --- |
| Risk of bias across studies | 15 | Specify any assessment of risk of bias that may affect the cumulative evidence (e.g., publication bias, selective reporting within studies). | 9 |
| Additional analyses | 16 | Describe methods of additional analyses (e.g., sensitivity or subgroup analyses, meta-regression), if done, indicating which were pre-specified. | 9 |
| **RESULTS** | | |  |
| Study selection | 17 | Give numbers of studies screened, assessed for eligibility, and included in the review, with reasons for exclusions at each stage, ideally with a flow diagram. | 9 |
| Study characteristics | 18 | For each study, present characteristics for which data were extracted (e.g., study size, PICOS, follow-up period) and provide the citations. | 10 |
| Risk of bias within studies | 19 | Present data on risk of bias of each study and, if available, any outcome level assessment (see item 12). | 11 |
| Results of individual studies | 20 | For all outcomes considered (benefits or harms), present, for each study: (a) simple summary data for each intervention group (b) effect estimates and confidence intervals, ideally with a forest plot. | 11 |
| Synthesis of results | 21 | Present results of each meta-analysis done, including confidence intervals and measures of consistency. | 11 |
| Risk of bias across studies | 22 | Present results of any assessment of risk of bias across studies (see Item 15). | 12 |
| Additional analysis | 23 | Give results of additional analyses, if done (e.g., sensitivity or subgroup analyses, meta-regression [see Item 16]). | 12 |
| **DISCUSSION** | | |  |
| Summary of evidence | 24 | Summarize the main findings including the strength of evidence for each main outcome; consider their relevance to key groups (e.g., healthcare providers, users, and policy makers). | 12-16 |
| Limitations | 25 | Discuss limitations at study and outcome level (e.g., risk of bias), and at review-level (e.g., incomplete retrieval of identified research, reporting bias). | 16 |
| Conclusions | 26 | Provide a general interpretation of the results in the context of other evidence, and implications for future research. | 17 |
| **FUNDING** | | |  |
| Funding | 27 | Describe sources of funding for the systematic review and other support (e.g., supply of data); role of funders for the systematic review. |  |

*From:*  Moher D, Liberati A, Tetzlaff J, Altman DG, The PRISMA Group (2009). Preferred Reporting Items for Systematic Reviews and Meta-Analyses: The PRISMA Statement. PLoS Med 6(7): e1000097. doi:10.1371/journal.pmed1000097

For more information, visit: **www.prisma-statement.org**.

**Supplementary Table 2.** Literature search strategies

| Database | Search strategies |
| --- | --- |
| MEDLINE | ((("college student"[Title/Abstract]) OR ("university student"[Title/Abstract]) OR ("undergraduate"[Title/Abstract]) OR ("higher education student"[Title/Abstract]) OR ("universit*"[Title/Abstract]) OR ("college*"[Title/Abstract])) AND (("fear"[Title/Abstract]) OR ("phobic disorders"[Title/Abstract]) OR ("panic disorder"[Title/Abstract]) OR ("concern"[Title/Abstract]) OR ("anxiet*"[Title/Abstract]) OR("Worr*"[Title/Abstract]) OR ("fear*"[Title/Abstract]))) AND (("2019-nCoV"[Title/Abstract]) OR ("SARS-CoV-2"[Title/Abstract]) OR ("2019 novel coronavirus"[Title/Abstract]) OR ("covid 19 virus"[Title/Abstract]) OR ("coronavirus disease 2019 virus"[Title/Abstract]) OR ("covid19 virus"[Title/Abstract]) OR ("2019 novel coronavirus disease"[Title/Abstract]) OR ("COVID19"[Title/Abstract]) OR ("covid 19 pandemic"[Title/Abstract]) OR ("sars cov 2 infection"[Title/Abstract]) OR ("covid 19 virus disease"[Title/Abstract]) OR ("2019 novel coronavirus infection"[Title/Abstract]) OR ("2019 ncov infection"[Title/Abstract]) OR ("coronavirus disease 2019"[Title/Abstract]) OR ("coronavirus disease 19"[Title/Abstract]) OR ("2019 ncov disease"[Title/Abstract]) OR ("covid 19 virus infection"[Title/Abstract])) |
| Embase | ('severe acute respiratory syndrome coronavirus 2':ab,ti OR 'sars-cov-2':ab,ti OR '2019 novel coronavirus':ab,ti OR 'covid-19 virus':ab,ti OR 'coronavirus disease 2019 virus':ab,ti OR 'covid19 virus':ab,ti OR '2019 novel coronavirus disease':ab,ti OR 'covid19':ab,ti OR 'covid-19 pandemic':ab,ti OR 'sars-cov-2 infection':ab,ti OR 'covid-19 virus disease':ab,ti OR '2019 novel coronavirus infection':ab,ti OR '2019-ncov infection':ab,ti OR 'coronavirus disease 2019':ab,ti OR 'coronavirus disease-19':ab,ti OR '2019-ncov disease':ab,ti OR 'covid-19 virus infection':ab,ti) AND ('university student':ab,ti OR 'college student':ab,ti OR 'undergraduate':ab,ti OR 'higher education student':ab,ti) AND ('fear*':ab,ti OR 'phobic disorders':ab,ti OR 'panic disorder':ab,ti OR 'concern':ab,ti OR 'anxiet*':ab,ti OR 'Worr*':ab,ti) |
| CINAHL | ((MJ college student OR MJ university student OR MJ undergraduate OR MJ higher education student) AND (MJ fear OR MJ panic disorder OR MJ phobic disorder OR MJ concern OR MJ anxiety OR MJ worry) AND ((TI severe acute respiratory syndrome coronavirus 2 OR AB severe acute respiratory syndrome coronavirus 2) OR (TI 2019 novel coronavirus disease OR AB 2019 novel coronavirus disease) OR (TI covid19 OR AB covid19) OR (TI 2019 novel coronavirus disease OR AB 2019 novel coronavirus disease)) |
| PsycINFO | AB (undergraduate OR university student OR college student OR higher education student) AND AB (fear OR panic disorder OR phobic disorder OR concern OR anxiety OR worry) AND AB ( COVID-19 OR severe acute respiratory syndrome coronavirus 2 OR SARS2 OR SARS-CoV-2 OR 2019 novel coronavirus OR COVID-19 virus OR coronavirus disease 2019 virus OR COVID19 virus OR Wuhan seafood market pneumonia virus OR 2019 novel coronavirus disease OR COVID19 OR COVID-19 pandemic OR SARS-CoV-2 infection OR COVID-19 virus disease OR 2019 novel coronavirus infection OR 2019-nCoV infection OR coronavirus disease 2019 OR coronavirus disease-19 OR 2019-nCoV disease OR COVID-19 virus infection) |

**Supplementary Table 3.** Univariate meta-regression analysis

| Variable | Estimate | Standard error | *P*-value |
| --- | --- | --- | --- |
| Mean age | -0.0027 | 0.2530 | 0.992 |
| Sample size | -0.0002 | 0.0005 | 0.738 |
